# Tuberculous otomastoiditis in children complicated by homolateral sigmoid sinus thrombosis: a case report

**DOI:** 10.11604/pamj.2024.48.100.44240

**Published:** 2024-07-12

**Authors:** Fadoua Ouzidan, Najib Benmansour, Mohammed Ridal, Mohamed Noureddine El Amine El Alami

**Affiliations:** 1 Department of Otolaryngology and Head and Neck Surgery, Faculty of Medicine and Pharmacy of Fes, Hassan II University Hospital, University Sidi Mohamed Ben Abdellah, Fes, Morocco

**Keywords:** Otomastoiditis, tuberculosis, diagnosis, case report

## Abstract

Tuberculosis is a major public health problem in Morocco and most of the developing countries but tuberculous otomastoiditis is quite rare. Here we report a case where a seven-year-old patient had chronic right otomastoiditis and retro-auricular fistula, whose specificity is its persistence after prolonged unsuccessful medical and surgical therapy for more than 2 months, and its complication by homolateral sigmoid sinus thrombosis. Computed Tomography (CT) scan of temporal bones showed complete destruction of right mastoid cells and a hypodense complement in the right middle ear. A right drainage through the retro auricular fistula with mastoid bone biopsy confirmed the tuberculous otomastoiditis diagnosis. The clinical and radiological outcome was favorable after anti-tuberculosis treatment for 6 months. Tuberculosis otomastoiditis is well described in the literature. However, its very low incidence often impedes consideration when faced with these latent infections. This is more so the case when concomitant pulmonary symptoms are absent. Thus, the importance of such publications is a reminder to think beyond the frequent diagnosis and prevent serious complications due to delayed treatment.

## Introduction

Morocco is a tuberculosis-endemic country [1]. Tuberculous otomastoiditis, a manifestation of tuberculosis affecting the ear and mastoid region, stands out for its diagnostic challenges due to its infrequency. In this article, we seek to present a case report of a patient with otomastoiditis tuberculosis whose late diagnosis resulted in the occurrence of a dangerous complication, in the hopes of enhancing clinical awareness and fostering a timelier intervention.

## Patient and observation

**Figure 1:**
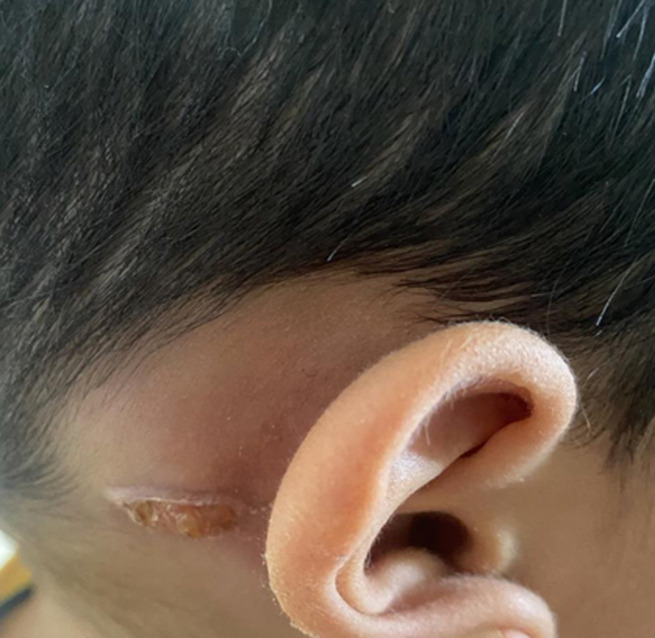
the appearance of a right retro-auricular swelling

**Patient information:** a 7-year-old child with no history of tuberculosis infection presented with a two-month history of right ear otorrhea. Two weeks later, the condition worsened with the development of a gradually increasing right retro-auricular swelling (Figure 1). The child experienced unremitting fever, general health decline, anorexia, and a weight loss of 5 kilograms over one month. Despite being treated with amoxicillin twice, there was no improvement. This prompted the parents to seek consultation at the pediatric emergency room, where an otorhinolaryngologist (ENT) doctor's opinion was solicited.

**Clinical finding:** the patient was found to be stable, with a normal temperature of 36.9°C, a respiratory rate of 37 breaths per minute, and a heart rate of 99 beats per minute. The child's weight was 19 kilograms and height was 123 centimeters. The otolaryngological examination revealed a soft, painful retro-auricular swelling measuring 3.5 cm, without any signs of inflammation (Figure 1).

**Figure 2:**
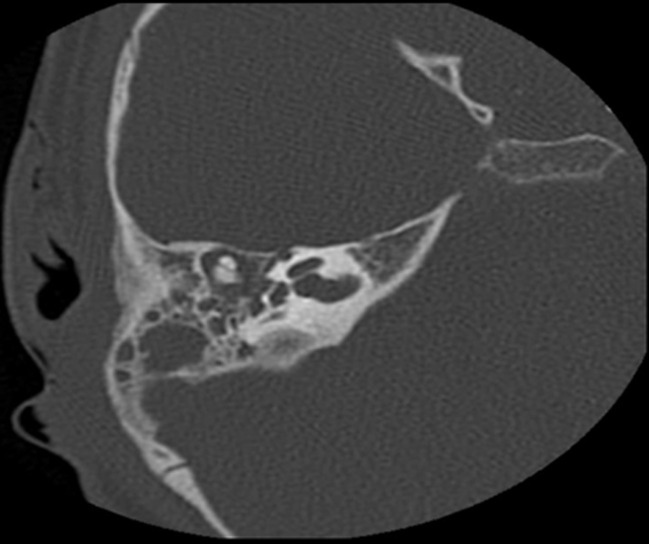
computed tomography scan of the craniofacial region showing tissue infiltration centered on the right retro-auricular region

**Figure 3:**
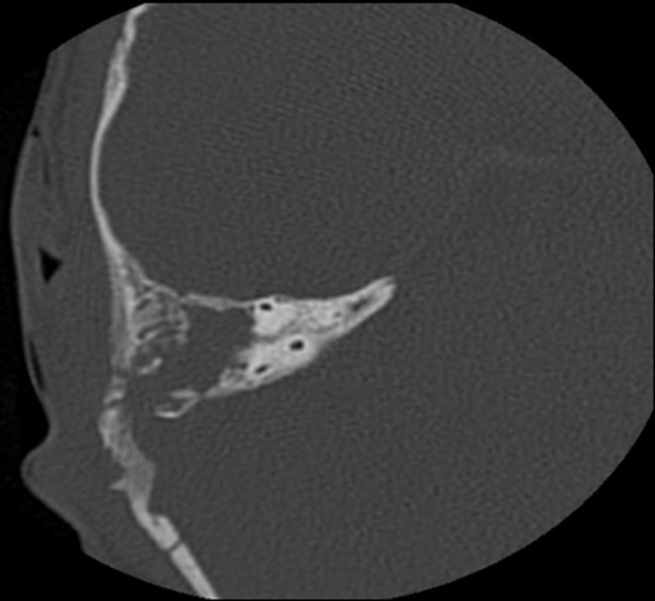
computed tomography scan of the craniofacial region showing bony lysis of the temporal bone and mastoid cells

**Diagnostic and assessment:** blood work was done which showed a high white blood cell count and an elevated C-reactive protein (CRP), and then a CT scan was performed, which showed: 1) tissue infiltration centered on the right retro-auricular region, measuring 40 x 35 x 40 mm in diameters, poorly defined, heterogeneously dense, with heterogeneous enhancement after contrast, outlining small confluent liquid collections within, the largest of which measures 14 x 6 mm in diameters (Figure 2); 2) bony lysis of the temporal bone and mastoid cells with intracranial extension and invasion of the ipsilateral sigmoid sinus, which presents a focal opacification defect suggestive of thrombosis of the right sigmoid sinus (Figure 3); 3) hypodense complement in the right middle ear, with no visualization of lysis of the ossicular chain. Chest X-ray was clear.

**Therapeutic intervention:** drainage and lavage of the mass were then performed under anesthesia in the operating room with a pus culture whose cyto-bacteriological study was negative. The patient was then hospitalized and put under triple injectable antibiotic therapy consisting of gentamicin for 5 days associated with cefixim and metronidale for 10 days without improvement. No anticoagulation was added due to the infectious nature of the thrombosis. Thus, the decision was made to admit the patient to the operating room, conduct decortication, and obtain a bone biopsy within the mastoid region whose histopathological study showed: granuloma showing epitheloid cells, Langhans giant cells, inflammatory infiltration, and caseous necrosis; all indicators of tuberculosis. The patient then was put under anti-tuberculosis chemotherapy for 6 months.

**Figure 4:**
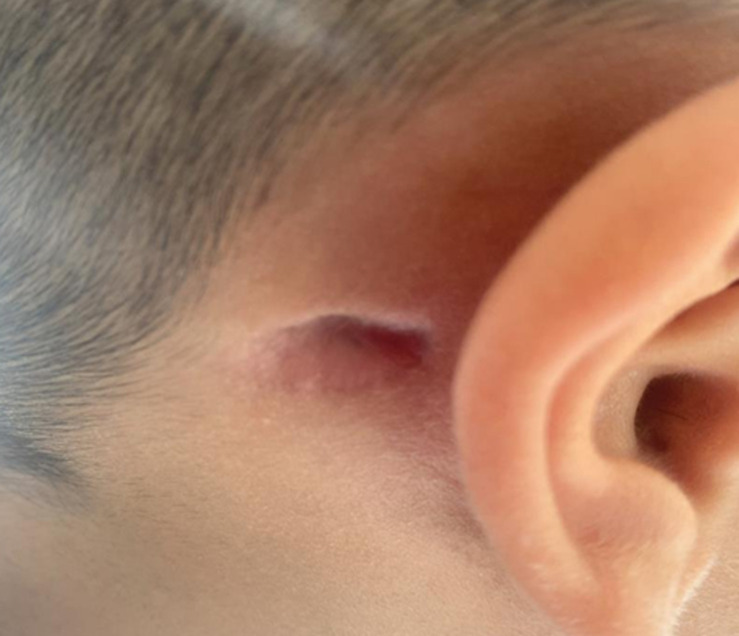
the disappearance of the right retro-auricular swelling

**Figure 5:**
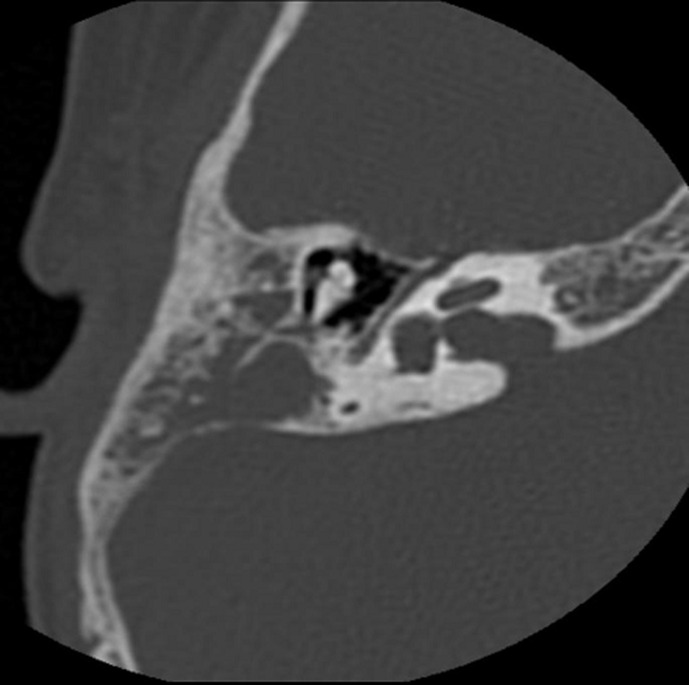
temporal bone scan showing reduction of tissue infiltration centered on the right retro-auricular region

**Follow-up and outcomes:** we note very favorable clinical and biological outcomes (Figure 4). The follow-up CT scan revealed: reduction of tissue infiltration centered on the right retro-auricular region (Figure 5). It is planned to regularly review this patient to screen for any potential recurrence.

**Parent´s perspective:** we were saddened and confused by the long time it took to get a specific diagnosis, and it was a big relief to see the improvement in his health after starting the anti-tuberculosis chemotherapy. Adam is gaining weight now and he is more active at school. We are very satisfied by this course of treatment.

**Informed consent:** the patient´s parents were informed of this publication, the importance of presenting such unusual presentations of tuberculosis, and its role in preventing complications due to misdiagnosis. They gave full written consent.

**Figure 6:**
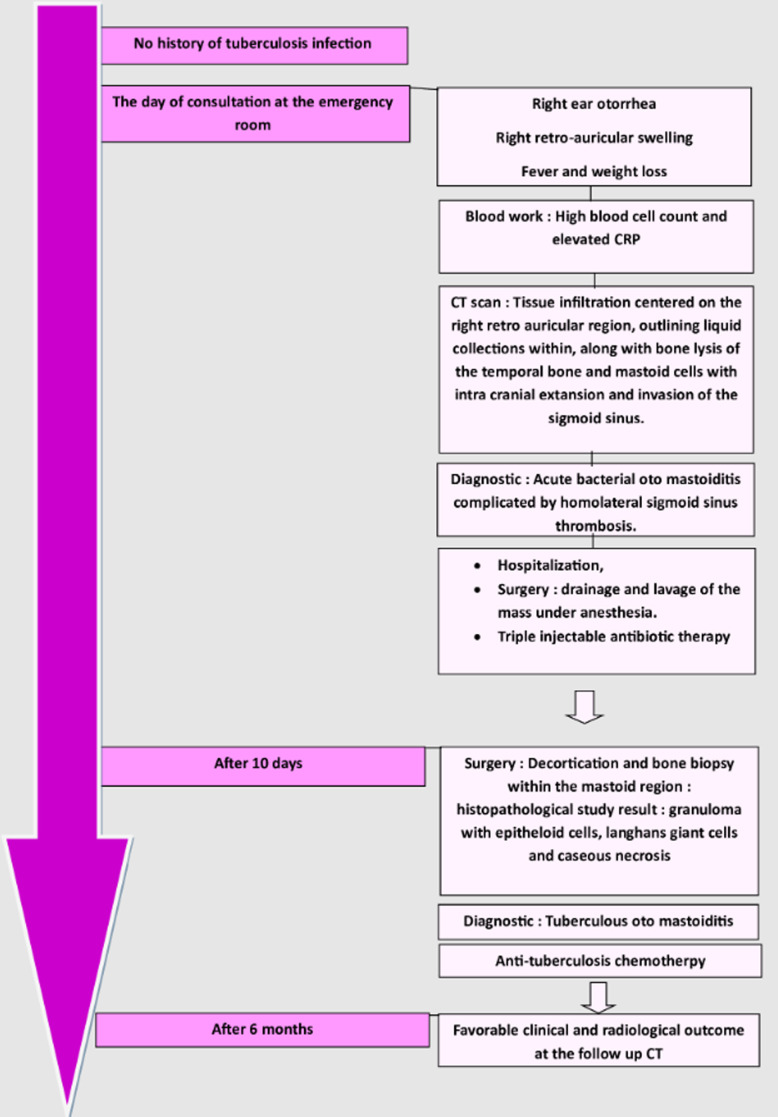
timeline of events

**Timeline of events:** see Figure 6.

## Discussion

*Mycobacterium tuberculosis* is identified as the causative agent in approximately 0.05% to 0.9% of cases of chronic middle-ear infections [2]. The majority of existing medical literature on tuberculous otomastoiditis originates from regions such as Asia, Africa, and Europe, where the prevalence of the disease is higher.

According to a study published in 2007 in Turkey [3], tuberculous otomastoiditis was identified in 11 out of 32 patients diagnosed with tuberculous meningitis, accounting for 34.3% of cases. In nine of these 11 patients, otomastoiditis was found on the same side as the meningeal involvement. Additionally, two patients exhibited bilateral otomastoiditis along with meningeal involvement.

In another investigation, Vaamonde *et al*. [2] in Spain diagnosed 10 cases between the years 1996 and 2002.

As for the United States of America, there were 11 documented cases of tuberculous otomastoiditis reported during the period spanning 1990 to 2003 [4]. This number is estimated to increase with the growing number of immunosuppressed patients, such as transplant recipients, those receiving tumor necrosis factor-alpha inhibitors, and those with human immunodeficiency virus infection [5].

According to the literature, significant delays in diagnosis, often spanning from 12 months to several years, are commonly reported due to the infrequent occurrence of this infection [1]. In our case, only 2 months delay was noted.

Tuberculous otomastoiditis should be taken into consideration when differentiating chronic otitis media, whether in individuals diagnosed with tuberculosis or those lacking evidence of tuberculosis in other areas. This becomes particularly pertinent when conventional medical treatments fail to improve persistent otorrhea.

In most cases, a clinical triad is observed, consisting of persistent otorrhea notwithstanding antibiotic treatment, facial paralysis (which alone can suggest a tubercular origin), and multiple tympanic perforations.

A few other cases have the particularity of presenting otalgia such as the case registered in Turkey by Bal *et al*.in 2012 [6], the series of Yang which noted the occurrence of pain in only 13% of patients [7] and the case reported in Marrakech that correlated the pain to the mastoid extension along with retroauricular swelling [1]. Another major symptom frequently highlighted in the literature is hearing loss [8]. The definitive confirmation of a tuberculous otomastoiditis diagnosis involves culturing M. tuberculosis from either the local discharge or a biopsy sample.

A positive indication from acid-fast staining using auramine and Ziehl-Neelsen techniques in otorrhea strongly suggests the presence of tuberculous otomastoiditis. In cases where acid-fast stains yield negative results, nucleic acid amplification techniques such as polymerase chain reaction (PCR) can offer valuable insights. Notably, the application of the real-time PCR test Xpert MTB/RIF (Cepheid, Inc., Sunnyvale, CA) to otorrhea samples has proven effective in identifying infections. Furthermore, the diagnosis may be supported by histopathological examination of tissue, along with observed improvement following specific anti-tuberculosis therapy [5].

While not conclusive for a definitive diagnosis, radiological examinations, such as CT scans, can reveal distinctive signs indicative of potential underlying conditions, such as tuberculosis affecting the middle ear and mastoid cavity when faced by an atypical chronic suppurative otitis media [1].

Upon confirming the diagnosis of tuberculous otomastoiditis, optimal treatment requires the collaborative expertise of an ear, nose, and throat surgeon and an infectious disease specialist. The preferred anti-tuberculosis therapy involves a combination of isoniazid and rifampin, complemented by pyrazinamide for the initial two months. Ethambutol is typically administered until the possibility of resistant M. tuberculosis is ruled out. The final treatment plan is individualized based on in vitro susceptibility studies.

While formal treatment trials lack sufficient data, recommendations are drawn from reported cases, advocating for a medical treatment duration ranging from 6 to 9 months [5]. Our patient received 6-month anti-tuberculosis chemotherapy with good evolution, therefore.

In addition to obtaining diagnostic tissue, the surgeon may contribute therapeutically by removing infected debris, as per our case, where the patient has undergone a drainage and a decortication later on. Surgical intervention becomes essential in the presence of complications such as facial nerve paralysis, sub-periosteal abscess, labyrinthitis, persistent post-auricular fistula, and infection extension into the central nervous system [9].

Instances where surgical intervention combined with chemotherapy have shown an accelerated recovery process for the affected ear [10]. Upon completing therapy, reconstructive procedures may be considered to improve hearing in specific patients.

## Conclusion

Tuberculous otomastoiditis exhibits clinical features that closely resemble chronic suppurative otomastoiditis, leading to potential confusion among clinicians. Distinguishing characteristics include persistent otorrhea, complete occupation of the tympanic cavity and mastoid air cells by soft tissue, and evidence of bone erosion or sequestra on CT scans. A meticulous inquiry into the patient's medical history, specifically regarding tuberculosis, is crucial to avoid misdiagnosis. While standard anti-tuberculosis chemotherapy remains the primary treatment for tuberculous otomastoiditis, cases involving surgical intervention alongside chemotherapy have demonstrated a faster healing process for the affected area.
